# In-Situ Modification of Nanofiltration Membranes Using Carbon Nanotubes for Water Treatment

**DOI:** 10.3390/membranes13070616

**Published:** 2023-06-21

**Authors:** Catalina Vargas-Figueroa, Luis Pino-Soto, Angelo Beratto-Ramos, Yesid Tapiero, Bernabé Luis Rivas, María Elizabeth Berrio, Manuel Francisco Melendrez, Rodrigo M. Bórquez

**Affiliations:** 1 Departamento de Ingeniería Química, Facultad de Ingeniería, Universidad de Concepción, Edmundo Larenas 219, Concepción 4070409, Chile; catavargas@udec.cl (C.V.-F.); aberatto@udec.cl (A.B.-R.); 2 Departamento de Polímeros, Facultad de Ciencias Químicas, Universidad de Concepción, Edmundo Larenas 129, Concepción 4070371, Chile; yesidtm@gmail.com (Y.T.); brivas@udec.cl (B.L.R.); 3 Advanced Nanocomposites Research Group (GINA), Departamento de Ingeniería en Materiales (DIMAT), Universidad de Concepción, Edmundo Larenas 315, Concepción 4070415, Chile; mariaberrio@udec.cl (M.E.B.); mmelendrez@udec.cl (M.F.M.)

**Keywords:** membrane filtration, carbon nanotubes, nanofiltration (NF) modification, Spielger-Kedem model, poptube

## Abstract

Modification of thin-film composite (TFC) nanofiltration (NF) membranes to increase permeability and improve separation performance remains a significant challenge for water scarcity. This study aimed to enhance the permeability and selectivity of two commercial polyamide (PA) NF membranes, NF90 and NF270, by modifying them with carbon nanotubes (CNTs) using microwave (MW)-assisted in-situ growth. The conducting polymer, polypyrrole (Ppy), and a ferrocene catalyst were used to facilitate the growth process. Chemical and morphological analyses confirmed that the surface of both membranes was modified. The NF270-Ppy-CNT membrane was selected for ion rejection testing due to its superior permeability compared to the NF90-Ppy-CNT. The modified NF270 membrane showed a 14% increase in ion rejection while maintaining constant water permeability. The results demonstrated that it is feasible to attach CNTs to a polymeric surface without compromising its functional properties. The Spliegler–Kedem model was employed to model the rejection and permeate flux of NF270-Ppy-CNT and NF270 membranes, which indicated that diffusive transport contributes to the modification to increase NaCl rejection. The present study provides a promising approach for modifying membranes by in-situ CNT growth to improve their performance in water treatment applications, such as desalination.

## 1. Introduction

Membrane-based separation technology has become increasingly popular for its use in wastewater treatment and drinking water production in recent years [1,2]. This is because membrane separation, especially polymeric membranes, has demonstrated numerous advantages, such as high separation efficiency, low energy consumption, modularity, easy adaptability, and low environmental impact [3,4,5,6]. In particular, nanofiltration (NF) membranes reject multivalent salts and organic molecules (>200 Da) [7], thereby rendering them an ideal technology for cost-effective seawater treatment with high efficiency compared to reverse osmosis (RO) [5,8,9]. The most common NF membrane is the thin-film composite (TFC) polyamide membrane [7,10], which acts as the active layer, and is supported by polysulfone (middle) and polyester (bottom). Although TFC membranes exhibit good separation performance, there is still interest in improving their properties, such as monovalent ion separation, without significantly sacrificing the water production permeability of the membrane.

Recently, methods to modify membranes with carbon nanotubes (CNTs) have attracted attention due to their unique properties, such as mechanical strength, chemical inertness, conductivity, and excellent water transport properties [11,12]. Surface modification with CNTs improves water permeability and increases antifouling properties and selectivity [11,13].

Generally, CNT membranes can be classified into two manufacturing categories: (1) vertically aligned CNT membranes (VA-CNT) and (2) mixed-CNT membranes (composites) [14,15]. For VA-CNT membranes, nanotubes are arranged vertically in a straight line, perpendicular to the membrane surface. In mixed-CNT membranes, pre-elaborated CNTs are embedded into a polymeric matrix such as polyamide (PA). A significant advantage of VA-CNT is that nanotube channels facilitate water flow through the tubes. However, their fabrication is complex to produce on a commercial scale [14,15,16]. On the other hand, mixed-CNT membranes have a relatively simple procedure, similar to the current elaboration of TFC membranes [17].

Mixed-CNT is mainly elaborated to improve the properties of ultrafiltration membranes. Previous studies report the incorporation of CNT suspensions by using techniques such as phase inversion [18,19], solution mixing [20,21], in-situ colloidal polymerization and precipitation [1,22,23,24], layer-by-layer [25], and interfacial polymerization [26,27]. These methods have been shown to improve filtration performance. However, some studies reported a significant decrease in membrane selectivity and mechanical stability. This result is due to decreased polymer cross-linking grade, a random distribution of CNTs in the active layer, and problems with microporous support adhesion. Moreover, these methods have shown low reproducibility and high processing costs [15,19,27,28].

In order to optimize membrane modifications by CNTs, the present study proposes a novel modification that allows the in-situ incorporation of CNTs into the membranes. This method, called Poptube, was developed by Zhang and Liu [29], and proposes the modification of a surface by employing a conductive polymer, a solid substrate, and microwave (MW) irradiation. This last is absorbed by a catalyst to increase the temperature locally and induce the growth of CNTs [30].

The synthesis process can be divided into (1) the development of catalyst particles and nucleation of CNTs; and (2) the growth and elongation of the fibers [30]. The growth of CNTs starts at the catalyst surface after the catalyst is saturated with carbon ions. The length and density of CNTs can be managed by multi-stage growth or by adding small organic molecules, such as hexane or pyridine [31]. Fe-C catalyst particles are lifted to the heads of the nanotubes. The ripening phenomenon of Ostwald can explain the dissipation of these catalyst particles, where catalyst particles can move through interdiffusion between atoms [31]. Conductive polymers have alternating double and single bonds. These conjugated structures have the property of having 𝜋 electronic orbitals extended throughout the network, i.e., electrons with freedom of movement that allow electricity to be conducted in the polymer chain [32].

The main difference with other techniques such as CNT-embedded polyamide TFC membrane via interfacial polymerization is that Poptube is faster and has a lower production cost. Other modification methods are complex and use expensive equipment, inert gas protection, vacuum system, or multiple serial processes [29,33,34]. This method has been applied to materials such as polyester (Kevlar), quartz, glass, and cement, but this is the first study that involved Poptube growth into filtration support [33,35,36].

Finally, the aim of this study was to perform in situ growth of carbon nanotubes (CNTs) on the surface of commercially available polyamide nanofiltration (NF) membranes. The modification was carried out using the conductive polymer polypyrrole (Ppy) and ferrocene as precursors for the rapid growth of CNTs. Ppy was selected due to its inherent properties as a biocompatible, inert, non-toxic, and stable material, making it a safe material for potable water generation. The obtained membrane was characterized in terms of surface morphology, chemistry, hydrophobicity, filtration performance, species rejection, and chemical composition.

## 2. Materials and Methods

### 2.1. Materials

Commercial TFC PA NF membranes NF90 and NF270 (Dow Chemical, Midland, MI, USA) were used. TFC membranes consist of an active layer, a microporous polysulfone interlayer, and a polyester support layer. The difference between these two TFC membranes is the composition of the active layer. Details of their properties can be found in previous research carried out by our research group [5].

Pyrrole (98%), ferric acid hexahydrate (97%), methyl orange, ferrocene (98%), ethanol (99.8%), methanol (99.8%), and HCl (37%) were used for in-situ carbon nanotube growth. All reagents were purchased from Sigma-Aldrich, St. Louis, MO, USA. Propylparaben has been used for pre-washing the membranes.

### 2.2. Synthesis In-Situ of Carbon Nanotubes

The membranes were pre-washed with propylparaben and then subjected to ultraviolet radiation for 30 s. Subsequently, the membranes were dried and washed with distilled water (9 µS/cm).

The synthesis of Ppy was evaluated by adapting previously defined methods by Bober et al. Bober, et al. [37], Zhao et al. Zhao, et al. [26], and Hazarika et al. Hazarika, et al. [33]. To investigate the formation of Ppy nanotubes, membranes were immersed in a solution of pyrrole in the presence of methanol, HCl or methyl orange for 2 h. Then, FeCl_3_ was added to form a final solution of 5 mM pyrrole, 5 mM methyl orange and 50 mM FeCl_3_. The solution was kept for 2 h with gentle agitation and then kept for 24 h at room temperature.

**Figure 1:**
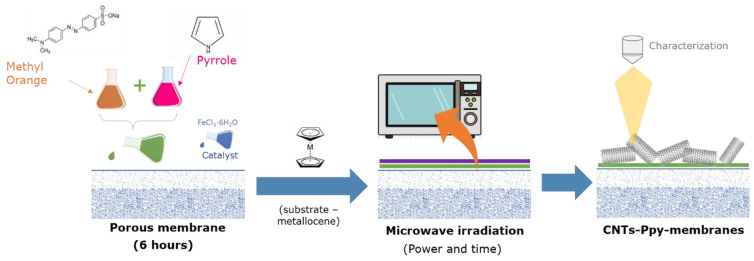
Schematic diagram of in-situ nanotube growth methodology on commercial membranes.

The synthesis of carbon nanotubes was performed using the methodology described by Zhang and Liu Zhang and Liu [29]. The metallocene was ferrocene (5 mM) with methanol as solvent. The membrane was impregnated for 30 min. Afterwards, the modified membrane was subjected to drying at 24 ± 1 °C to eliminate any residual solvent. Subsequently, it was irradiated using a MW inverter (LG Smart inverter, Beijing, China) at 200 W for a duration of 1 min (Figure 1).

### 2.3. Characterization of Membrane Surface

The surface morphology changes were characterized by scanning electron microscopy (SEM) with the JEOL JSM-6390LV equipment at a voltage of 20 kV. The samples were previously coated with a gold target using a Sputter Coater 50/60 Hz SPI brand (Wate Chester, PA, USA). The roughness of the membranes was analyzed with an atomic force microscope (AFM; AC OmegaScope 1000 mode AIST-NT Inc., Novato, CA, USA) using a non-contact method. The hydrophilicity of the membrane surface was measured through contact angle experiments (KRÜSS DSA25S droplet shape analyzer, Hamburg, Germany) using 10 µL of deionized water to measure the contact angles according to the sessile droplet technique. The Wenzel Equation [38] was used to eliminate the contribution of roughness in this measurement.

(1) $$\mu_{obs}=\cos\left(\phi \right)=r\cos\left(\theta \right)$$

where cos(θ) as the intrinsic wettability and *r*, as the roughness factor or Wenzel factor, is defined as the ratio of the actual area to the projected area of the surface, which can be obtained with AFM analysis.

Raman spectroscopy with the NT-MDT equipment (model NTEGRA Spectra) was used as a non-destructive technique to evaluate the deposits made with a 633 nm laser.

### 2.4. Measurement of Water Permeability

All filtration tests were performed in a high-pressure stirred cell (Sterlitech HP4750, Auburn, WA, USA) with an active membrane area of $1{.46\;\times \;10}^{-3}$ m^2^ (Figure S1). The experiments were performed at 24 °C and 1200 rpm, and before each experiment, the membranes were washed with distilled water and compacted for 1 h at 30 bar.

The permeate flux density is calculated according to Equation (2).

(2) $$J_{w}=\frac{\Delta V}{A_{m}\cdot \Delta t}\;$$

where Δ*V*/Δ*t* is the permeate volume over time and $A_{m}$ is the effective filtration area. The hydraulic permeability constant ($k_{w}$) was determined using the following expression:

(3) $$k_{w}=\frac{J_{w}}{\Delta P}\;$$

where ΔP is the transmembrane operating pressure difference. The following expression calculates the observed rejection of the solute:

(4) $$R_{ob}\;\left(\%\right)=\left(1-\frac{C_{ps}}{C_{fs}}\right)\times 100$$

where $C_{fs}$ is the conductivity of the solute in the feed, and $C_{ps}$ is the conductivity of the solute in the permeate. The transport of solutes through a nanofiltration membrane can be described using the principles of non-equilibrium thermodynamics, treating the membrane as a black box. The Spiegler–Kedem (SK) model has been widely used to characterize the membrane in terms of two unique parameters: the reflection coefficient (*σ*) and solute permeability ($P_{s}$). In a two-component system consisting of a solute and water, where $J_{w}$ and $J_{s}$ represent the water and solute fluxes, respectively, it can be described as [39]:

(5) $$J_{w}=k_{w}\cdot \left(\Delta P-\sigma \cdot \Delta \pi \right)$$

(6) $$J_{s}=P_{s}\cdot \Delta C_{s}+\left(1-\sigma \right)\cdot J_{w}\cdot C_{ms}$$

where Δπ is the osmotic pressure difference of the solution, $\Delta C_{s}=C_{ms}-C_{ps}$, and $C_{ms}$ is the solute concentration at the membrane surface. According to Equation (6), the solute flux is the sum of the diffusive and convective terms. The osmotic pressure difference (Δπ) can be calculated using Vant–Hoff’s equation:

(7) $$\Delta \pi =\frac{R_{g}\;T}{m}\left(C_{ms}-C_{s}\right)$$

where $R_{g}$ is the ideal gas constant, T is the temperature, and m is the molar mass of the solute. According to the Spielger–Kedem model, the permeability coefficient ($P_{s}$) and the reflection coefficient (σ) can be obtained by solving the following equations [39]:

(8) $$R_{R}=\sigma \frac{\left(1-F\right)}{\left(1-\sigma \cdot F\right)}$$

(9) $$F=exp\;\left(-\frac{1-\sigma }{P_{s}}\cdot J_{w}\right)$$

where F is a dimensionless parameter and $R_{R}$ is the actual solute rejection. The actual rejection ($R_{R}$) can also be defined as:

(10) $$R_{R}\;\left(\%\right)=\left(1-\frac{C_{ps}}{C_{ms}}\right)\times 100$$

The Spielger–Kedem model relates the solute concentration on the membrane to the solute concentration in the permeate, requiring the incorporation of concentration polarization. Therefore, it is necessary to combine the model with film theory using a correlation that allows for the determination of the mass transfer coefficient ($k_{s}$). This transfer coefficient depends on factors such as feed velocity, temperature, and geometry. The relationship between the solute concentration at the surface and in the permeate can be expressed as [6]:

(11) $$\frac{\left(C_{ms}-C_{ps}\right)}{\left(C_{fs}-C_{ps}\right)}=\exp\left(\frac{J_{v}}{k_{s}}\right)$$

where $k_{s}$ is defined as:

(12) $$k_{s}=\frac{D_{sw}}{\delta }$$

where $D_{sw}$ is the diffusion coefficient of the solute and δ is the concentration polarization layer thickness. On the other hand, the mass transfer coefficient is related as follows:

(13) $$k_{s}=\frac{Sh\cdot D_{sw}}{r_{sc}}$$

where $r_{sc}$ is the radius of stirred cell and Sh is the Sherwood number [40]:

(14) $$Sh=0.285\cdot Re^{0.55}\;Sc^{0.33}$$

Reynolds number:

(15) $$Re=\frac{\rho \cdot \omega \cdot r_{sc}^{2}}{\mu }$$

Schmidt number:

(16) $$Sc=\frac{\mu }{\rho \cdot D_{sw}}$$

Combining both models, the observed rejection can be expressed as [41]:

(17) $$\frac{R_{ob}}{1-R_{ob}}=\frac{\sigma }{1-\sigma }\left(1-\exp\left(\frac{-J_{v}\;\left(1-\sigma \right)}{P_{s}}\right)\right)\left(\exp\left(-\frac{J_{v}}{k_{s}}\right)\right)$$

NaCl was the monovalent ion used as a representative in this study to evaluate the rejection performance of the modified membranes. The tests are performed on the same stirred cell equipment. After pressurizing with distilled water for 1 h at 30 bar, the water was replaced with a NaCl solution (1000 mg/L). The tests were performed over a wide pressure range to represent real operating conditions.

## 3. Results and Discussions

### 3.1. Synthesis, Morphology, and Physicochemical Characterization of Modified Membranes and Carbon Nanotubes

In-situ carbon nanotube synthesis was performed using the Poptube technique; therefore, it does not require excessively high temperatures, unlike the chemical vapor deposition technique [29]. For PA TFC membranes, time and maximum power in the MW oven were fixed at 200 W and 60 s to avoid explosive reactions and damage to the membrane surface (Figure S2).

**Figure 2:**
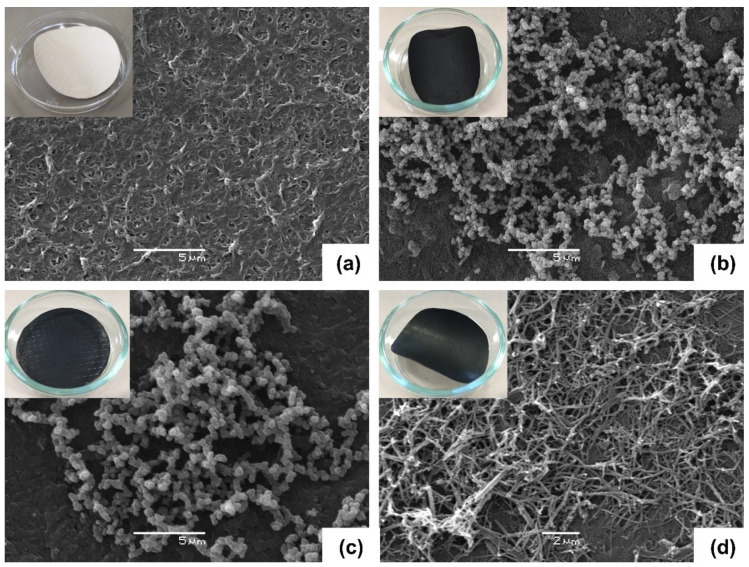
SEM images of (**a**) unmodified NF90, (**b**) NF90-Ppy in the presence of methanol, (**c**) HCl and (**d**) methyl orange. Inserts correspond to the macroscopic view of membranes.

The use of Ppy as a conducting polymer and its polymerization on the surface of membranes could allow a more homogeneous distribution for the subsequent growth of CNTs. The synthesis of Ppy was evaluated in the presence of methanol, HCl, and methyl orange. SEM images show that Ppy acquires different morphologies. While in the presence of methanol and HCl, Ppy polymerizes in globular form, methyl orange shows a nanotube conformation (Figure 2).

As mentioned in previous studies, pyrrole, upon oxidation in the presence of methyl orange, polymerizes in Ppy nanotubes rather than in a globular form [37,42]. The morphology changes from globular to 1D nanotubes or nanofibers are possible due to polymerization conditions and the introduction of additives in the reaction. In this case, methyl orange synthesizing Ppy produces a well-defined 1D nanotubular morphology with a high aspect ratio, high conductivity, and good environmental stability. The acid-base transition of the methyl oxide salt appears to be associated with the formation of 1D objects [42]. Compared to the globular structure, the Ppys one-dimensional form has a significant advantage that improves conductivity and possesses a lower percolation threshold. This conformation contributes to better heat distribution, generating a more homogeneous distribution of carbon nanotubes on the surface [37,42,43].

Many parameters count in the encapsulation of iron nanoparticles inside CNTs, among which are: the viscosity of the fused nanoparticles, the frictional force between the inner walls of the CNTs and the trapped iron nanoparticles, capillary force of the nanotubes during their growth, and the tension between the different forces and pressure on the iron nanoparticles due to the growth of the nanotubes [33].

**Figure 3:**
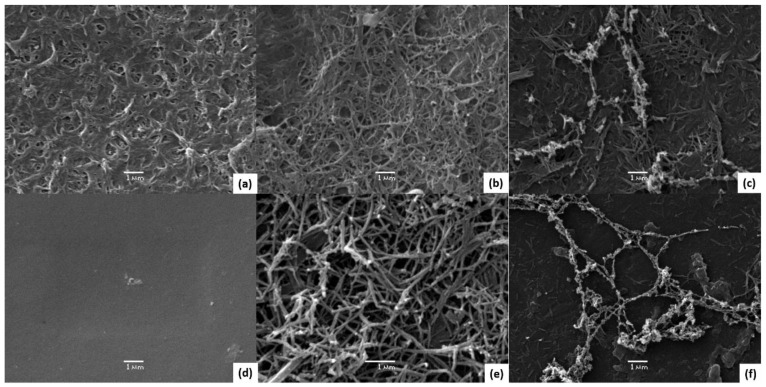
SEM images of modified membranes. (**a**) Unmodified NF90, (**b**) NF90-Ppy, (**c**) NF90-Ppy-CNTs, (**d**) unmodified NF270, (**e**) NF270-Ppy and (**f**) NF270-Ppy-CNTs.

SEM surface characterization of unmodified membranes (NF90 and NF270), membranes with Ppy deposition (NF90-Ppy and NF270-Ppy), and membranes with carbon nanotubes (NF90-Ppy-CNTs and NF270-Ppy-CNTs) are shown in Figure 3. The results show that in Figure 3b,e the Ppy was polymerized in the form of nanotubes for NF90 and NF270 membranes. Subsequently, the addition of ferrocene develops the synthesis of CNTs on a layer of Ppy (Figure 3c,f). Finally, the synthesis of CNTs on the membrane is evident; additionally, the growth of nanostructures on a thin polymeric material (~2 mm) met a significant challenge, considering the high probability of substrate ignition at a substantial temperature change of the reaction.

Roughness measurements were performed with scan sizes of 10 × 10 µm^2^ to compare the values of RMS (root mean square) and Ra (mean roughness) of virgin commercial membranes NF270 and NF90 (Table 1 and Figure S3). The analysis of membrane roughness revealed that NF270-Ppy-CNT exhibited a substantial increase in both the root mean square value (127%) and average roughness (155%) compared to NF270. In contrast, NF90-Ppy-CNT showed a comparatively smaller increase in the root mean square value (3%) and average roughness (1%) compared to NF90 (Table 1). According to Wang, et al. Wang, et al. [44], the most significant intermolecular interaction between CNTs and the aromatic compounds on the membrane surface is the pi-pi type interaction. These findings align with previous studies on the chemical structure of NF270 and NF90 membranes [45,46], which established that the NF270 membrane offers more available interaction sites due to its uncoated semi-aromatic structure. Another contributing factor to the increased modification of NF270 over NF90 is the stiffness of the NF90 membrane compared to the NF270 membrane, primarily due to its fully aromatic nature.

The contact angles of unmodified NF90 and NF270 were 55.66 ± 1.78 and 32.77 ± 2.80, respectively, while the modified membranes were 81.02 ± 0.94 and 74.87 ± 0.44, for NF90-CNTs and NF270-CNTs, respectively (see Table 1 and Figure S4). Contact angles showed significant changes (45 and 128%) compared to unmodified membranes with small concentrations of Ppy, which could be due to the incorporation of hydrophobic CNTs. Shawky, et al. Shawky, et al. [20], and Wang, et al. Wang, et al. [47] reported similar results when they incorporated CNTs into porous membranes. However, increasing the CNTs’ concentration for a mixed-CNT generally cause a decrease in contact angle, increasing permeability and slightly sacrificing ion rejection [15,19,22,24,25,26,27,28]. Previous studies have demonstrated that rough surfaces exhibit higher contact angles due to surface irregularities that impede liquid movement, resulting in droplets being suspended on surface protrusions [48]. These findings suggest that the observed reduction in surface energy following the modification is likely a result of nanotube supersaturation, which contributes to increased surface roughness [49,50]. On the other hand, the heterogeneous rough surface causes the water droplet to be deposited on the nanotube and not on the active surface of the membrane, resulting in a more hydrophobic surface than the original one [47,50]. Vatanpour et al. Vatanpour, et al. [51] observed that increasing the concentration of oxidized multiwalled carbon nanotubes (MWCNTs) coated with Ppy on a polyvinylidene fluoride (PVDF) ultrafiltration membrane caused an increase in the contact angle. Its results were related to the increase in surface roughness due to the agglomeration of MWCNTs on the surface.

**Figure 4:**
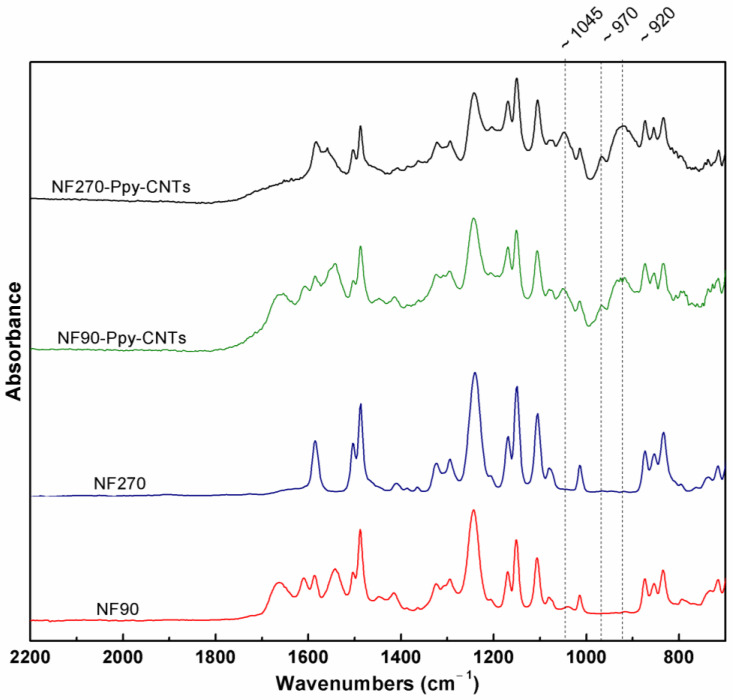
FTIR-ATR spectra of virgin and modified membranes.

The ATR-FTIR spectra of the membrane and the selected modifications are shown in Figure 4. The analysis range used reflects both the PA active layer and the polysulfone support layer of commercial membranes since the FTIR signal has a relatively deep penetration (>300 nm) [45]. Therefore, the performed analysis of the chemical modification of the surface is mainly performed on the most relevant signals. The commercial membranes NF90 and NF270 present peaks between 1545 and 1650 cm^−1^ of secondary amine, aromatic amide, amide I, and amide II vibrations. On the other hand, the modified membranes present peaks at wavenumbers ~920, ~970, and ~1045 cm^−1^, related to C-O-C groups, N-H stretching vibration, and C-H in-plane deformation, respectively. These results demonstrate the presence of Ppy and CNT oxidation reactions [43,52]. It is important to note that polyamide bands only suffer small changes in their positions and intensities with nanotube incorporation. This indicates that the reaction forms a thin layer that does not obstruct the original support, unlike other CNT modifications [37].

**Figure 5:**
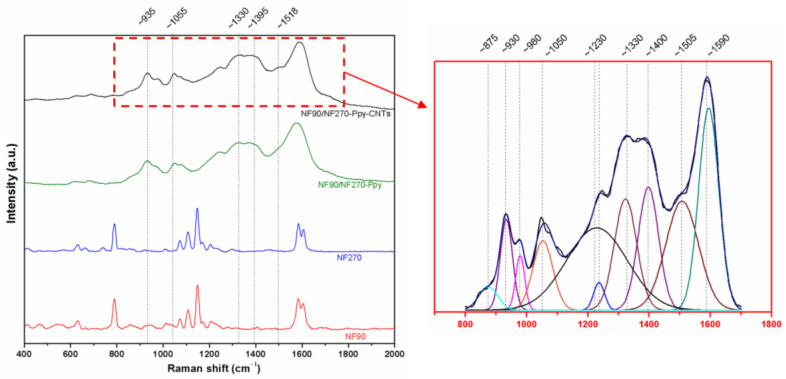
RAMAN spectrum of the membrane modified by in-situ carbon nanotube synthesis. Right plot corresponding to deconvolution of NF90/NF270-Ppy-CNTs spectra in detail.

To further confirm the changes in the functional groups of the membranes before and after modification, a RAMAN spectrum characterization was performed. In comparison with FTIR, the RAMAN spectrum showed noticeable changes (Figure 5). Modified membranes exhibited signals around 920, 989, 1043, and 1410 cm^−1^, characteristic of the Ppy. The signals at 920 and 989 cm^−1^ correspond to ring deformation associated with dication (bipolarons) and radical cations (polarons), respectively. Finally, the band observed at 1043 cm^−1^ is attributed to in-plane C-H deformation and the signal around 1410 cm^−1^ to C-N stretching of the pyrrole ring [53].

The RAMAN signals, corresponding to the membrane and the carbonaceous materials of Ppy and CNTs, overlap. Due to this, a deconvolution of the signals using Lorentzian line shapes of the red delimited area in Figure 5 was performed. Carbon nanotubes present three typical bands in the RAMAN spectrum for 1.96 eV (633 nm) around 1324 cm^−1^ (D band), 1606 cm^−1^ (G band) associated with the in-plane vibration of the C-C bond, and around 2646 cm^−1^ (G’ band) attributed to the overtone of the D band [54]. The position and intensity of the D and G’ bands depend on the wavelength of the exciting laser line. The ratio of the intensities of the D and G bands ($I_{D}/I_{G}$) results in 0.55, which is similar to 0.59 found in previous studies for CNT synthesis from ferrocene [55].

### 3.2. Filtration and Ion Rejection Performance of Modified Membranes

**Figure 6:**
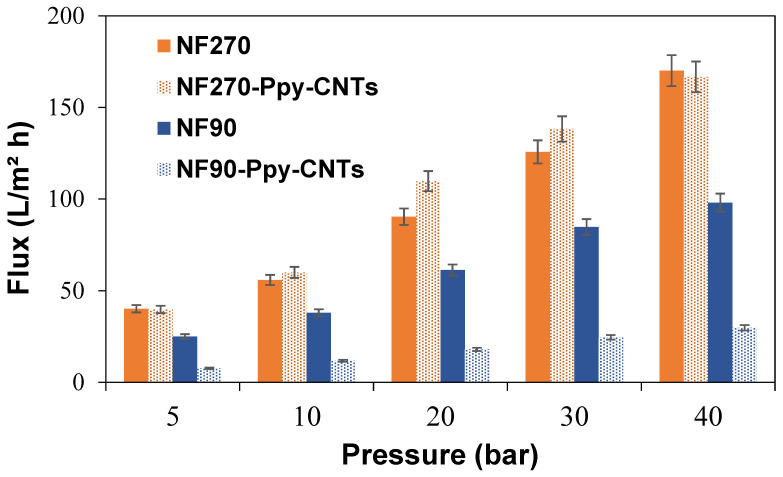
Permeate flux density as a function of operating pressure for the modified NF270 and NF90 membranes.

Water permeability experiments aim to demonstrate the capacity and efficiency of filtration through the membrane. The results obtained for both modified membranes are shown in Figure 6. The increase in permeate flux with increasing operating pressure agrees with other publications [56,57]. It is important to note that the modified membranes allow working pressures typical of actual desalination plant operations. This is one of the advantages of working with commercial membranes as structural support. Most studies in VA-CNT test their filtration experiments below 8 bar due to the lack of mechanical stability to resist the typical operating pressures of desalination plants [14,15,16].

NF90 showed a pure water flux of 2.13 ± 0.39 L/m^2^ h bar, due to its small pore size (r_pore_ = 0.34 nm). The pure water flux value of the NF90-Ppy-CNTs membrane was reduced to 0.63 ± 0.18 L/m^2^ h bar, due to pore blockage by the unreacted Ppy on the surface [58,59]. For the NF270 membrane (r_pore_ = 0.42 nm), the pure water flux value was 3.68 ± 0.42 L/m^2^ h bar, while NF270-PPy-CNTs showed a similar value (3.67 ± 0.83 L/m^2^ h bar). In this case, pore blockage was compensated by the increased roughness of NF270-PPy-CNTs, which enhanced the filtration area and enhanced pure water flux [19]. Therefore, NF270-PPy-CNTs were selected for salt rejection studies.

**Figure 7:**
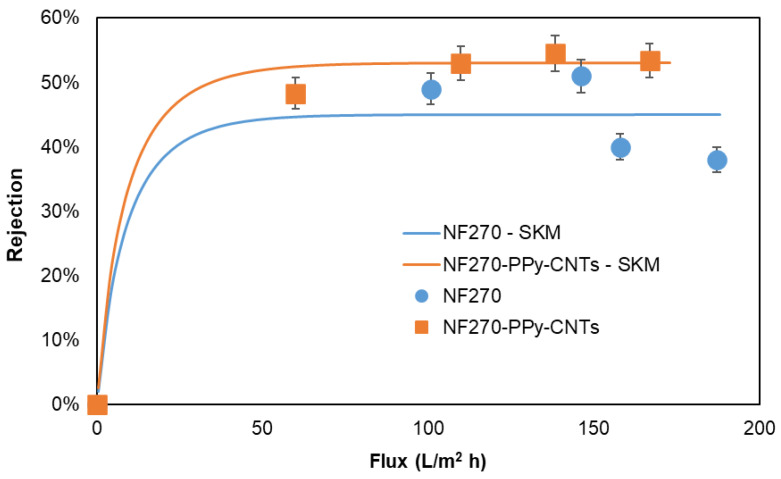
Solute retention against permeate flux curve from experimental data and predicted results from the Spielger–Kedem model for NF270 and NF270-Ppy-CNTs.

The rejection capacity of the NF270-Ppy-CNTs membrane is slightly higher than that of the NF270 commercial membrane, as shown in Figure 7. The findings of this study demonstrate that the introduction of CNTs into the commercial NF270 membrane yields a comparable behavior to that observed in tight NF membranes [40]. Specifically, the membrane rejection exhibited a consistent value that remained unaffected by the escalating water flux permeability. This is in stark contrast to the behavior observed in loose membranes, as exemplified by the NF270, where an increase in water flux permeability results in a reduction in rejection efficiency. Probably, the surface pores of the modified NF270 are blocked by the PPy-CNTs, which causes the rejection ability to increase, enhancing steric repulsion [18]. In the case of water permeability, Figure 7 shows that there is a difference between the values of NF270 and NF270-Ppy-CNT that decreases with increasing permeate flux. In the highest permeability case, NF270-Ppy-CNT showed a permeate flux reduction of 10.83% compared to NF270. Therefore, the membrane modification by Ppy-CNT improves the rejection properties from 38.4% to 53.4% while slightly sacrificing the product water yield. Additionally, the experimental data on the observed rejection of NaCl as a function of permeate flux were fitted using the Spielger–Kedem model to determine the values of the reflection coefficient and solute permeability (Figure 7 and Table 2). The fitting of the experimental data with the Spielger–Kedem model shows a good correlation between the calculated values and the experimental values. This allows for an analysis of the ion transport behavior between the NF270 and NF270-Ppy-CNT membranes. The reflection coefficient σ of NF270-Ppy-CNTs increases slightly, indicating that ion transport occurs more through diffusion than convection.

## 4. Conclusions

This study demonstrated the possibility of in-situ cultivation of CNTs on the surface of commercial PA TFC membranes. The thermal process involved in Poptube modification does not damage the membrane surface.

The incorporation of Ppy and CNTs in commercial membranes successfully modified the properties of both membranes. The contact angle of both membranes increased due to the presence of Ppy-CNTs. The increased roughness of NF270-Ppy-CNTs compensated for the pore blocking caused by the incorporation of Ppy-CNTs. Unlike other studies, the growth of nanotubes on the NF270 membrane slightly increased NaCl rejection performance without excessively sacrificing membrane permeability. The Spiegler–Kedem model confirmed that it is possible to adjust the experimental data for both membranes, determining the predominant transport mechanism in filtration. In this case, NF270-Ppy-CNTs slightly enhanced ion transport through diffusion rather than convection compared to the commercial NF270 membrane. The modification achieved in the NF membranes through in-situ CNT growth assisted by MW enables the generation of PA membranes with improved separation properties in a simple, fast, and cost-effective manner to enhance water treatment applications. Future studies should focus on the growth technique to further improve the separation properties of the membranes and evaluate their anti-fouling properties.

## Figures and Tables

**Table 1:** Root mean square, average roughness, and contact angle of virgin and CNT-modified membranes.

Membrane	RMS Roughness [nm]	Ra [nm]	Contact Angle, Θw [°]
NF90-virgin	90.22	71.42	55.66 ± 1.78
NF270-virgin	35.00	24.90	32.77 ± 5.8
NF90-Ppy-CNT	93.19	70.84	81.02 ± 0.94
NF270-Ppy-CNT	79.46	63.46	74.87 ± 0.44

**Table 2:** Parameters estimated based on the experimental results for NF270 and NF270-Ppy-CNT.

Membrane	σ (-)	P (m s^−1^)	k (m s^−1^)
NF270	0.45	2.14 × 10^−6^	0.011
NF270-Ppy-CNTs	0.53	2.01 × 10^−6^	0.011

## Data Availability

The data presented in this study are available on request from the corresponding author. The data are not publicly available due to patent pending.

## Supplementary Materials

The following supporting information can be downloaded at: https://www.mdpi.com/article/10.3390/membranes13070616/s1, Figure S1: Filtration system used for modified membrane testing; Figure S2: Images of membrane damage due to overpowering, Figure S3: AFM images for (a) NF90, (b) NF270, (c) NF90-Ppy-CNTs and (d) NF270-Ppy-CNTs, Figure S4: Contact angle images for (a) NF90, (b) NF270, (c) NF90-Ppy-CNTs and (d) NF270-Ppy-CNTs.
