# Supplementary material for: In-Situ Modification of Nanofiltration Membranes Using Carbon Nanotubes for Water Treatment
Source: Membranes (Basel). 2023 Jun 21;13(7):616. doi: 10.3390/membranes13070616 (PMC10385991; doi:10.3390/membranes13070616)
Supplement: Supplementary file 1 [file membranes-13-00616-s001.zip › membranes-2430974-supplementary.pdf]

**Supplementary Material**

# In-Situ modification of nanofiltration membranes using carbon nanotubes for water treatment

**C. Vargas-Figueroa[1], L. Pino-Soto*[1], A. Beratto-Ramos[1], Y. Tapiero[2], B. L. Rivas[2], M.E. Berrio[3], M.F. Meléndrez[3] and R. Borquez[1]*.**

[1]Departamento de Ingeniería Química, Facultad de Ingeniería, Universidad de Concepción, Edmundo Larenas 219. Concepción, 4070409, Chile
[2] Departamento de Polímeros, Facultad de Ciencias Químicas, Universidad de Concepción, Edmundo Larenas 129. Concepción, 4070371, Chile.
[3] Advanced Nanocomposites Research Group (GINA). Departamento de Ingeniería en Materiales (DI-MAT), Universidad de Concepción, Edmundo Larenas 315. Concepción 4070415, Chile.

* Correspondence: luispino@udec.cl (L.P-S); rborquez@udec.cl (R.B.)

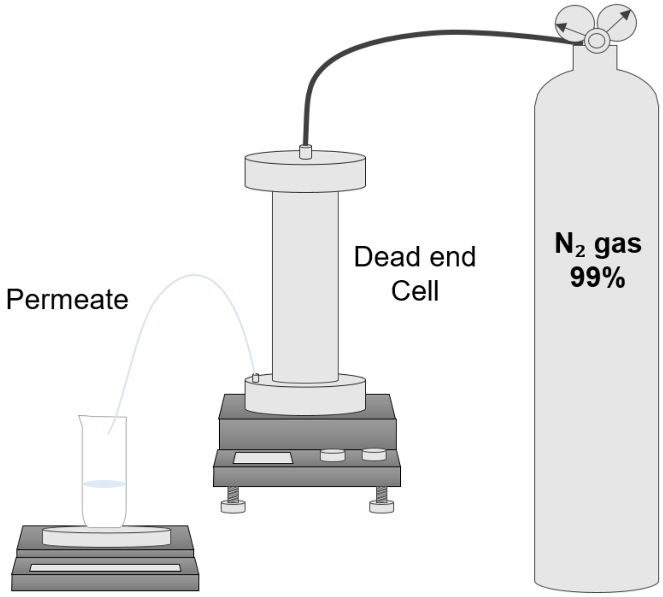


**Figure S1:** Filtration system used for modified membrane testing

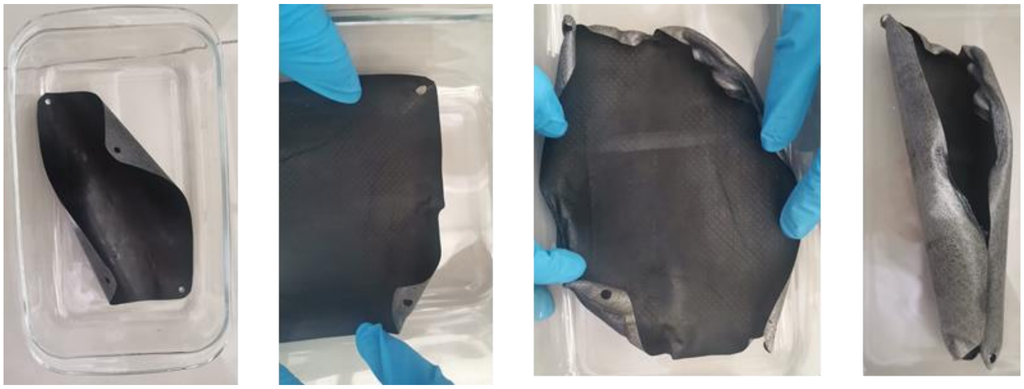

**Figure S2:** Images of membrane damage due to overpowering.

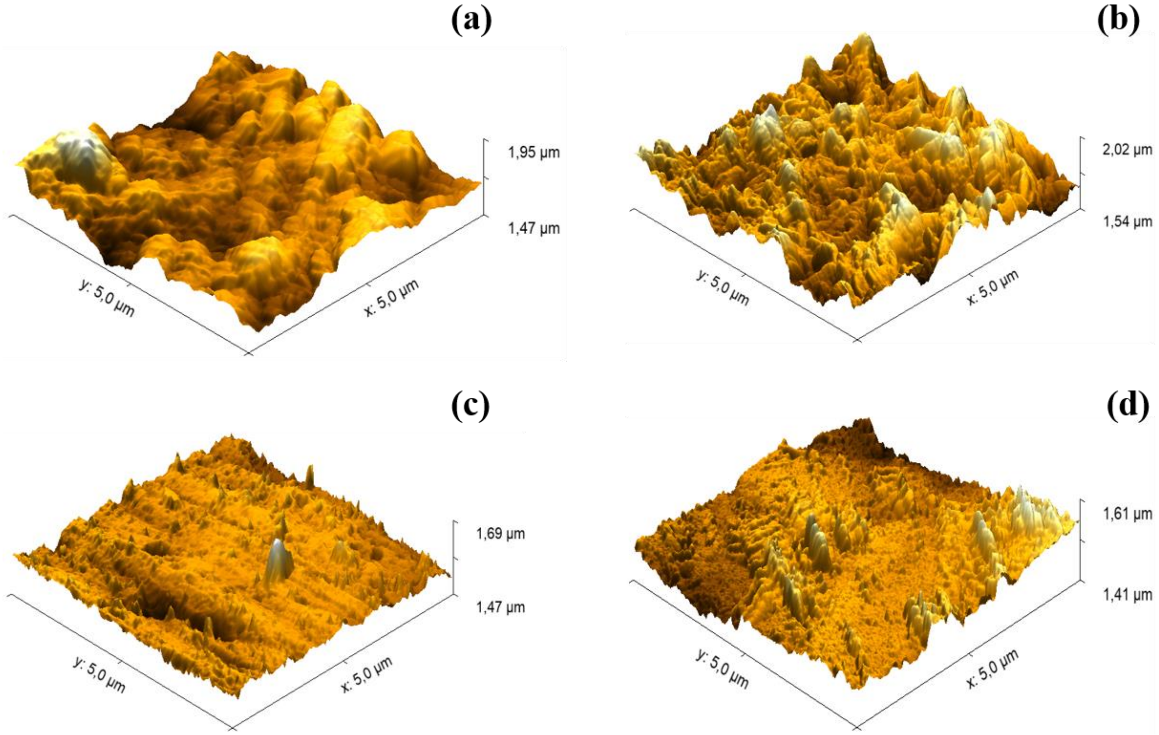


**Figure S3:** AFM images for (a) NF90, (b) NF270, (c) NF90-Ppy-CNTs and (d) NF270-Ppy-CNTs.

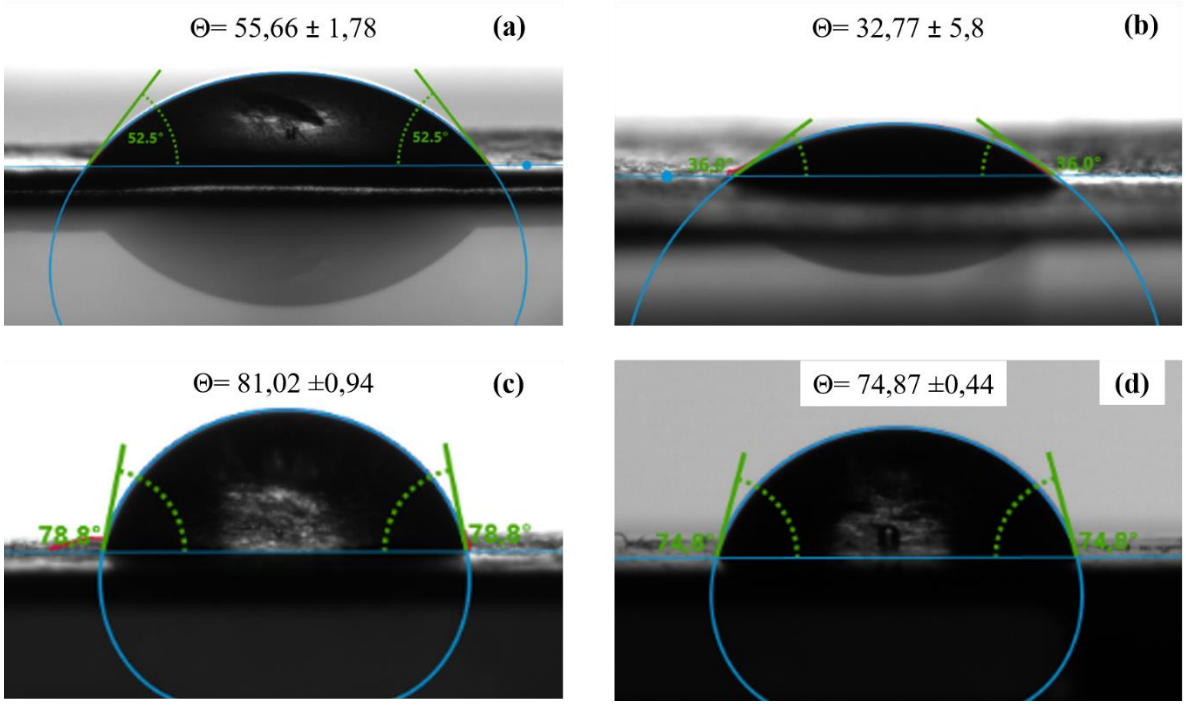


**Figure S4:** Contact angle images for (a) NF90, (b) NF270, (c) NF90-Ppy-CNTs and (d) NF270-Ppy-CNTs.
